# Intestinal Serotonin Transporter Inhibition by Toll-Like Receptor 2 Activation. A Feedback Modulation

**DOI:** 10.1371/journal.pone.0169303

**Published:** 2016-12-29

**Authors:** Eva Latorre, Elena Layunta, Laura Grasa, Marta Castro, Julián Pardo, Fernando Gomollón, Ana I. Alcalde, José E. Mesonero

**Affiliations:** 1 Departamento Farmacología y Fisiología, Facultad de Veterinaria, Instituto de Investigación Sanitaria de Aragón (IIS), Universidad de Zaragoza, Zaragoza. Spain; 2 RNA—Mediated Mechanisms of Disease, Institute of Biomedical and Clinical Sciences, University of Exeter Medical School. Exeter. United Kingdom; 3 Instituto Agroalimentario de Aragón–IA2- (Universidad de Zaragoza–CITA), Zaragoza, Spain; 4 Departamento Bioquímica y Biología Molecular y Celular, Facultad de Ciencias, Instituto de Investigación Sanitaria de Aragón (IIS), Universidad de Zaragoza, Zaragoza, Spain; 5 Servicio de Sistema Digestivo. Hospital Clínico Universitario "Lozano Blesa", Instituto de Investigación Sanitaria de Aragón (IIS); Centro de Investigación Biomédica en Red de Enfermedades Hepáticas y Digestivas (CIBEREHD), Zaragoza, Spain; Duke University School of Medicine, UNITED STATES

## Abstract

TLR2 is a microbiota recognition receptor that has been described to contribute to intestinal homeostasis and to ameliorate inflammatory intestinal injury. In this context, serotonin (5-HT) has shown to be an essential intestinal physiological neuromodulator that is also involved in intestinal inflammatory diseases. Since the interaction between TLR2 activation and the intestinal serotoninergic system remains non-investigated, our main aim was to analyze the effect of TLR2 on intestinal serotonin transporter (SERT) activity and expression and the intracellular pathways involved. Caco-2/TC7 cells were used to analyze SERT and TLR2 molecular expression and SERT activity by measuring 5-HT uptake. The results showed that apical TLR2 activation inhibits SERT activity in Caco-2/TC7 cells mainly by reducing SERT protein level either in the plasma membrane, after short-term TLR2 activation or in both the plasma membrane and cell lysate, after long-term activation. cAMP/PKA pathway appears to mediate short-term inhibitory effect of TLR2 on SERT; however, p38 MAPK pathway has been shown to be involved in both short- and long-term TLR2 effect. Reciprocally, 5-HT long-term treatment yielded TLR2 down regulation in Caco-2/TC7 cells. Finally, results from *in vivo* showed an augmented intestinal SERT expression in mice *Tlr2*^*-/-*^, thus confirming our inhibitory effect of TLR2 on intestinal SERT *in vitro*. The present work infers that TLR2 may act in intestinal pathophysiology, not only by its inherent innate immune role, but also by regulating the intestinal serotoninergic system.

## Introduction

Intestinal epithelium contributes to intestinal physiology and homeostasis, not only by acting as a physical barrier between the body and the microorganisms present in the lumen (microbiota), but also by carrying out an active participation in the mucosal immune response [1, 2]. The intestinal innate immune system recognizes specific microorganism-associated molecular patterns (MAMPs), in part by involving Toll-like receptors (TLRs). Intestinal epithelial cells express these receptors, which enable the epithelium to discriminate between commensal and pathogen microorganisms. Thus, the activation of TLRs generates a response either to tolerate or to eliminate the microorganism, depending on its recognition as either commensal or pathogen, respectively [3]. In addition, TLRs activity deregulation has been described as one of the main triggering events that cause inflammatory bowel diseases (IBDs) [4, 5].

Immunological activity mediated by TLRs protects and maintains the integrity of the mucosal barrier, thus contributing to the homeostasis and intestinal physiology. In this context, recent results have demonstrated that TLR2 may play an important role in intestinal homeostasis, and variants in the TLR1/2/6 genes have been associated with different phenotypes of IBDs [6]. TLR2 is localized on the cell surface and recognizes Gram-positive and mycobacterial MAMPs including bacterial lipopeptide, lipoteichoic acid, peptidoglycan and soluble tuberculosis factor [7]. An important feature of TLR2 activity is that it requires the co-expression of either TLR1 or TLR6, since two heterodimeric forms TLR2/1 or TLR2/6 are responsible for TLR2 activation [8]. TLR2 has been shown to contribute to epithelial barrier function by different mechanisms, including the organization of tight junction zonula occludens 1 protein (ZO-1) [9], the inhibition of intestinal epithelial cells apoptosis [10], the increase of intestinal mucosa repair and renewal [11] or the stimulation of the expression of mucus layer components [12]. Considering its activities on epithelial barrier function, TLR2 signaling has been postulated to ameliorate intestinal injury induced by chronic inflammatory processes.

In addition to the defensive barrier function, an important enteroendocrine activity arises from intestinal epithelium. In this context, 5-HT, which has been described as an essential intestinal neuromodulator, is mainly synthesized by enterochromaffin cells located in the intestinal epithelium. 5-HT regulates the whole intestinal physiology [13–15] and has also been demonstrated to be involved in intestinal inflammatory processes [16, 17]. 5-HT activity depends on the extracellular 5-HT availability that is mainly modulated by the serotonin transporter (SERT) expressed in the enterocytes. SERT is responsible for the 5-HT uptake into these cells, finishing 5-HT effects. Alterations in the activity of the intestinal serotoninergic system have been demonstrated to contribute to the origin and/or consolidation of chronic gastrointestinal diseases such as inflammatory bowel diseases (IBDs) [18]. Moreover, the 5-HT level has been shown to be altered in experimental intestinal inflammation and in IBD patients [19], and high levels of 5-HT have also been described in several inflammatory and diarrheal conditions [20]. In this context SERT activity has been described as being regulated by pro- and anti-inflammatory factors [17, 21, 22].

Recent results have suggested that TLRs may regulate the neuroendocrine activity of the intestinal epithelium [23, 24]. In relation to TLR2, previous results have shown that TLR2, TLR1 and TLR6 are co-expressed in human and mouse intestine and co-localize with 5-HT [25], suggesting a potential role for enteroendocrine cells in innate immune response through TLR activation. However, the interaction between TLR2 and the serotoninergic system remains unknown. Therefore, the aim of the present work was to analyze the effect of TLR2 activation on intestinal SERT activity and expression, and, reciprocally, to determine whether 5-HT modulates TLR2 expression. To carry out the present study, the human enterocyte-like Caco-2/TC7 cell line and intestine from *Tlr2*^*-/-*^ mice were used.

## Materials and Methods

### Reagents and antibodies

The following drugs and substances were used (respective suppliers in parentheses): Serotonin (5-hydroxytryptamine, 5-HT), selective p38MAPK inhibitor SB 220025, selective PKA inhibitor KT 5720 and selective inhibitor of ERK pathway PD98059, (Sigma–Aldrich; St. Louis, MO, USA). Pam3CSK4 and Pam2CSK4, specific TLR2/1 and TLR2/6 agonists respectively (InvivoGen; San Diego, CA, USA). [^3^H]-5-HT (specific activity 25–30 Ci/mM) (Perkin-Elmer; Boston, MA, USA). Primary antibodies used were: goat polyclonal antibody anti-human and anti-mouse SERT (ab130130) and rabbit monoclonal anti-human TLR2 (ab108998) (Abcam, Cambridge, UK); mouse monoclonal anti-human p38 (sc-7972), rabbit polyclonal antibody anti-human pp38 (sc-7975-R) and secondary antibodies coupled to horseradish peroxidase (Santa Cruz Biotechnology, Santa Cruz, CA, USA). All generic reagents were purchased from Sigma–Aldrich and Roche Applied Sciences (Sant Cugat del Vallés, Barcelona, Spain).

### Cell culture

This study was carried out in the human enterocyte-like cell line Caco-2/TC7 [26]. This cell line expresses SERT endogenously and has been described as an excellent intestinal model to study SERT activity and expression [27]. Caco-2/TC7 cells were cultured at 37°C in an atmosphere of 5% CO_2_ and maintained in high glucose DMEM supplemented with 2 mM glutamine, 100 U/ml penicillin, 100 μg/ml streptomycin, 1% non-essential amino acids, and 20% heat-inactivated FBS (Life Technologies, Carlsbad, CA, USA).

For 5-HT uptake assays, cells were seeded in 24-well plates at a density of 4 × 10^4^ cells per well, and uptake measurements were carried out 14 days after seeding (9 days after confluence). Previous results have shown that SERT activity reaches a plateau on the fifth day after confluence [27]. Cell medium was free of FBS 1 day before using the cells in the experiments. Pam3CSK4 and Pam2CSK4 (TLR2/1 and TLR2/6 ligands, respectively), and the different modifiers were added to the culture medium at different concentrations and periods, depending on the experiment. Preliminary assessment of the cell monolayer morphology was carried out in the cell culture under the different experimental conditions. The results have shown that none of the assayed treatments seemed to affect cell morphology, proliferation, and monolayer integrity (data not shown).

### Animals

Inbred C57BL/10 (wild type, WT) and a mouse strain deficient for TLR2 (*Tlr2*^*-/-*^) were bred at the at the Centro de Investigación y Tecnología Agroalimentaria (CITA, Zaragoza, Spain). Their genotypes were periodically analyzed as described [28]. Mice of 10–12 weeks of age were used in the experiments (both male and female). All mice were housed under pathogen-free conditions on a 12-hour light/dark cycle with food and water *ad libitum*. The experiments were approved by the Ethic Committee for Animal Experiments of the Zaragoza University. The care and use of animals were performed accordingly with the Spanish Policy for Animal Protection RD53/2013, which meets the European Convention for the Protection of Vertebrate Animals used for Experimental and other Scientific Purposes (Council of Europe No 123, Strasbourg 1985) and the European Union Directive 2010/63/EU on the protection of animals used for scientific purposes. Mice were euthanized by cervical dislocation. Immediately after death, the intestinal tract (ileum and colon) was removed and rinsed with an ice-cold solution of 0.9% NaCl. Tissue samples for RNA analysis were collected and preserved in RNAlater (Qiagen, Hilden, Germany) 1 day at 4°C, and then frozen at -80°C. Tissue samples for protein analysis were immediately frozen in ice isopropyl alcohol and stored at -80°C.

### 5-HT uptake studies

Uptake measurements were performed on cells attached to 24-well plates either under control conditions or after treatment with specific TLR2/1 or TLR2/6 ligands. The method used has been described elsewhere [27]. The transport medium composition was as follows: 137 mM NaCl, 4.7 mM KCl, 1.2 mM KH_2_PO_4_, 1.2 mM MgSO_4_, 2.5 mM CaCl_2_, 10 mM HEPES pH 7.4, 4 mM glutamine, 0.1% BSA, and both 0.2 μM 5-HT and [^3^H]-5-HT (1.5 μCi/ml) as substrate. Before measuring uptake, cells were pre-incubated at 37°C in an atmosphere of 5% CO_2_ with substrate-free transport medium for 30 min. The cells were immediately washed with substrate-free transport medium at 37°C and then incubated with transport medium at 37°C for 6 min. Transport was stopped by removing the transport medium and washing the cells twice with ice-cold, substrate-free transport medium containing 20 μM 5-HT. The cells were solubilized in 0.1 N NaOH, and samples were taken for radioactivity counting (Wallac Liquid Scintillation Counter, Perkin-Elmer), and protein measurement using the Bradford method (Bio-Rad, Hercules, CA) and BSA as a standard. Results were calculated in pmol 5-HT/mg protein and were expressed as a percentage of control value (100%). In the kinetic study, 5-HT uptake was measured in the concentration range 0.05–10 μM, and the kinetic constants *V*_max_ and *K*_t_ were calculated.

5-HT fluxes were measured by a method previously described [23]. Briefly, Caco-2/TC7 cells were seeded in 12 well permeable polyester (PET) membranes with porous size 0.4 μm and a 1 cm^2^ growth area (Millipore, Billerica, MA). These inserts established apical (A) and basal (B) compartments. Apical to basal (A-B) 5-HT fluxes were measured at intervals of 10 min during 1 h, after adding 0.1 μM 5-HT plus [^3^H]-5-HT (2.5 μCi/ml) to the apical compartment. After 20 min of equilibration period, samples were taken from the basal compartment every 10 min, and replaced with fresh medium. The results were calculated in pmol 5-HT/10 min and were expressed as a percentage of the control value (100%). Cell monolayer integrity and confluence were checked by measuring transepithelial resistance (TER) with an Epithelial Voltohmmeter (Millicell Electrical resistance system, Millipore) before beginning each experiment.

### RNA extraction, reverse transcription and real-time PCR

Total RNA was extracted from both Caco-2/TC7 cells cultured in 25 cm^2^ flasks (14 days after seeding) and mouse intestinal tissue. For intestinal RNA extraction, mouse tissue samples were thawed in an ice-cold RTL buffer (Qiagen) and homogenized using the Ultra Turrax T25 (IKA, Staufen, Germany). Lysates from intestinal tissue and Caco-2/TC7 cells were transferred in a QIAshredder column (Qiagen), and RNA was purified using the RNeasy mini kit (Qiagen), following the manufacturer’s instructions. Residual DNA was removed by an additional on-column DNase I digestion step using the Qiagen RNase-free DNase set (Qiagen).

The extracted RNA (1 μg) was used as a template for first-strand cDNA synthesis using oligo(dT) primers and a reverse transcriptase (Life Technologies). Negative amplification control was performed in the absence of reverse transcriptase. cDNAs obtained by reverse transcription (RT) were used to determine SERT and TLR2 mRNA expression levels. In Caco-2/TC7 human cells, SERT mRNA was measured using hSERT Gene Expression Assay from Applied Biosystems (Life Technologies, Assay number Hs00169010_m1 [SLC6A4]), with hGAPDH (Assay number Hs99999905_m1) and hHPRT1 (Assay number Hs99999909_m1) housekeeping gene expression used as calibrators (Life Technologies). Quantification of SERT mRNA in mice and TLR2 mRNA in Caco-2/TC7 cells was carried out by using SYBR green and the following specific primers: mSERT sense (5’ GGCAACATCTGGCGTTTTCC 3’), mSERT antisense (5’ ATTTCGGTGGTACTGGCCCA 3’), hTLR2 sense (5’ GAAGCTCCAGCAGGAACATC 3’), and hTLR2 antisense (5’ GAATGAAGTCCCGCTTATGAAGACA 3’). The corresponding housekeeping gene expression used as calibrators were in human, hGADPH (sense 5’ CATGACCACAGTCCATGCCATCACT 3’ and antisense 5’ TGAGGTCCACCACCCTGTTGCTGTA 3’) and hHPRT (sense 5’ CTGACCTGCTGGATTACA 3’ and antisense 5’ GCGACCTTGACCATCTTT 3’); and in mouse, mGADPH (sense 5’ AACGACCCCTTCATTGAC 3’and antisense 5’ TCCACGACATACTCAGCAC 3’) and mHPRT (sense 5’ CTGGTGAAAAGGACCTCTCGAA 3’ and antisense 5’ CTGAAGTACTCATTATAGTCAAGGGCAT 3’).

Each sample was run in triplicate, and the mean Ct was determined from the three runs. Relative mRNA expression under each experimental condition (control or treatment) was expressed as ΔCt = Ct_gene_−Ct_calibrator_. Then, relative mRNA expression was calculated as ΔΔCt = ΔCt_control_−ΔCt_treatment_. Finally, the relative gene expression levels were converted and expressed as fold difference (= 2^-ΔΔCt^).

### Preparation of Caco-2/TC7 brush border-enriched fraction and mouse intestinal samples for western blotting

Caco-2/TC7 cells were cultured in 75 cm^2^ flasks and used 14 d after seeding. The enterocyte brush border membrane-enriched fraction was obtained by divalent cation precipitation and differential centrifugation procedure described in a previous paper [29]. Briefly, cells were washed twice with PBS and immediately re-suspended with a cold Tris-mannitol buffer (2 mM Tris, 50 mM Mannitol, pH 7.1) containing protease inhibitors and 0.02% sodium azide. Mouse intestinal samples (ileum and colon) were thawed and homogenized using ultra-turrax in Tris-mannitol buffer pH 7.1, containing protease inhibitors and 0.02% sodium azide, and then the samples were progressively disrupted by using Potter-Elvehjem with a PTFE pestle. In both cases, the suspension was homogenized and disrupted by sonication (fifteen 1-s bursts, 60W). One sample was taken from the Caco-2/TC7 cells and mouse intestine lysates for total protein analysis and protein quantification.

For the preparation of brush border-enriched fraction from Caco-2/TC7 cells, 20 mM CaCl_2_ was added to the cell lysate and, after standing for 10 min on ice, the mixture was centrifuged for 10 min at 950 g. The supernatant was taken and centrifuged at 33500 g for 30 min. The pellet (brush border-enriched fraction) was re-suspended in phosphate buffer (10 mM KH_2_PO_4_/K_2_HPO_4_ pH 6.8), and a sample was taken for protein analysis. Protein concentration was measured using the Bradford method.

Brush border-enriched fraction and cell lysate from Caco-2/TC7 cells, and ileum and colon homogenates from WT and *Tlr2*^*-/-*^ mice (60 μg of total protein) were electrophoresed on 8% SDS-PAGE gels and then transferred to PVDF membranes by electroblotting. The membranes were blocked with 4% non-fat dried milk plus 1% BSA and probed with a goat polyclonal antibody anti-human or anti-mouse SERT (1:500), rabbit monoclonal anti-human TLR2 (1:1000), rabbit polyclonal antibody anti-human pp38-MAPK (1:500), or mouse monoclonal anti-human p38-MAPK (1:500). Primary antibodies were detected using specific secondary antibodies coupled with horseradish peroxidase and the ECL Plus detection kit (GE Healthcare, Buckinghamshire, UK) and were visualized with VersaDoc™ (Imaging System, Bio-Rad). After stripping, membranes were re-probed with goat polyclonal anti-human or anti-mouse β-actin to determine differences in the sample loading. The SERT/β-actin protein ratio was calculated in densitometric units from the film using Quantity One Analysis Software (Bio-Rad).

### Statistical analysis

All results are expressed as means ± the standard error of the mean (SE). Statistical comparisons were performed using one-way ANOVA, followed by the Bonferroni post-test with a confidence interval of 95% (*p* < 0.05). Normal distribution was previously confirmed with the D’Agostino-Pearson test. Analysis of the transport values obtained in the kinetic study of the 5-HT transport was performed by non-linear regression, fitting the results to an equation containing a saturable (Michaelis-Menten) plus a non-saturable (diffusion) component. The equation used was as follow: V = ((Vmax [5-HT]) / (Kt + [5-HT])) + (Kd [5-HT])). Statistical analysis was carried out with the computer-assisted Prism GraphPad Program (Prism version 4.0, GraphPad Software, San Diego, CA).

## Results

### The effect of TLR2 activation on 5-HT-uptake

**Fig 1:**
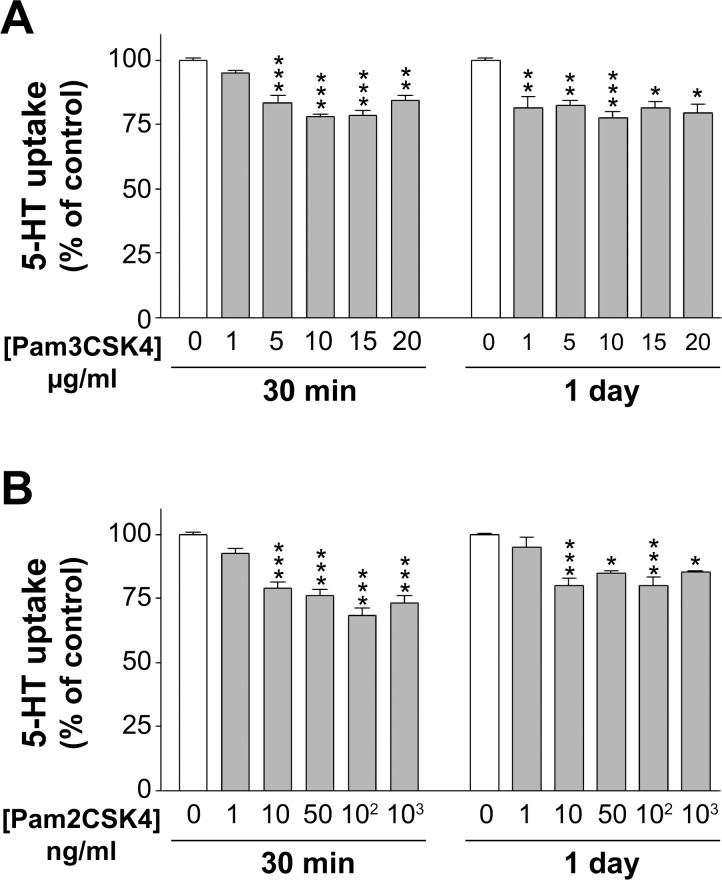
Effect of Pam3CSK4 (TLR2/1 ligand) and Pam2CSK4 (TLR2/6 ligand) on 5-HT uptake. Uptake was measured after 6 min incubation of 0.2 μM 5-HT. (A) Pam3CSK4 concentrations assayed were 1, 5, 10, 15, and 20 μg/ml. (B) Pam2CSK4 concentrations were 1, 10, 50, 100 and 1000 ng/ml. The treatment periods were 30 min (short-term) or 1 day (long-term). The results are expressed as the percentage of the uptake control (100%) and are the mean ± SEM of 5 independent experiments. Absolute control values were 11.05±0.50 and 10.90±0.63 pmol 5-HT/mg protein at 30 min or 1 day, respectively. ***P<0.001, **P<0.01, and *P<0.05 compared with the control value (untreated cells).

Preliminary analysis has demonstrated that Caco-2/TC7 cells expressed TLR2, TLR1, and TLR6 (data not shown). In order to analyze the effect of TLR2 activation on SERT activity, 5-HT uptake in Caco-2/TC7 cells was measured in cells treated with Pam3CSK4 (TLR2/1 ligand) or Pam2CSK4 (TLR2/6 ligand) at different concentrations and periods of treatment (30 min and 1 day). As shown in Fig 1, both TLR2/1 and TLR2/6 activation yielded a significant decrease (about 25% of reduction) of 5-HT uptake under the different conditions assayed.

In order to characterize these TLR2 effects on SERT, the kinetic study of the 5-HT transport was carried out, and kinetic constants *V*_max_ and *K*_*t*_, which indicate the capacity and affinity of SERT respectively, were calculated. 5-HT uptake was measured at different 5-HT concentrations (ranged 0.05–5 μM) in Caco-2/TC7 cells treated with 5 μg/ml Pam3CSK4 or 50 ng/ml Pam2CSK4 for 30 min or 1 day. The results have shown that the treatment of cells with either TLR2/1 or TLR2/6 ligands inhibited 5-HT uptake by mainly affecting *V*_max_ (SERT capacity) at both short (30 minutes) and long-term (1 day). However, none of the treatments seemed to affect transport affinity (*K*_*t*_) (Table 1).

**Table 1:** Kinetic constants of 5-HT uptake in Caco-2/TC7 cells after TLR2 activation. The results are the mean ± SEM of four experiments. *p<0.05 compared with control (untreated cells).

Time	Conditions	*V*_max_ (pmol 5-HT/mg protein)	*K*_t_ (μM)
**30 minutes**	**Control**	22.36±0.08	0.64±0.08
	**TLR2/1**	17.55±0.80*	0.72±0.02
	**TLR2/6**	11.5±0.01*	0.64±0.06
**1 day**	**Control**	29.5±0.96	0.75±0.08
	**TLR2/1**	20.26±0.61*	0.61±0.06
	**TLR2/6**	20.25±0.74*	0.74±0.07

**Fig 2:**
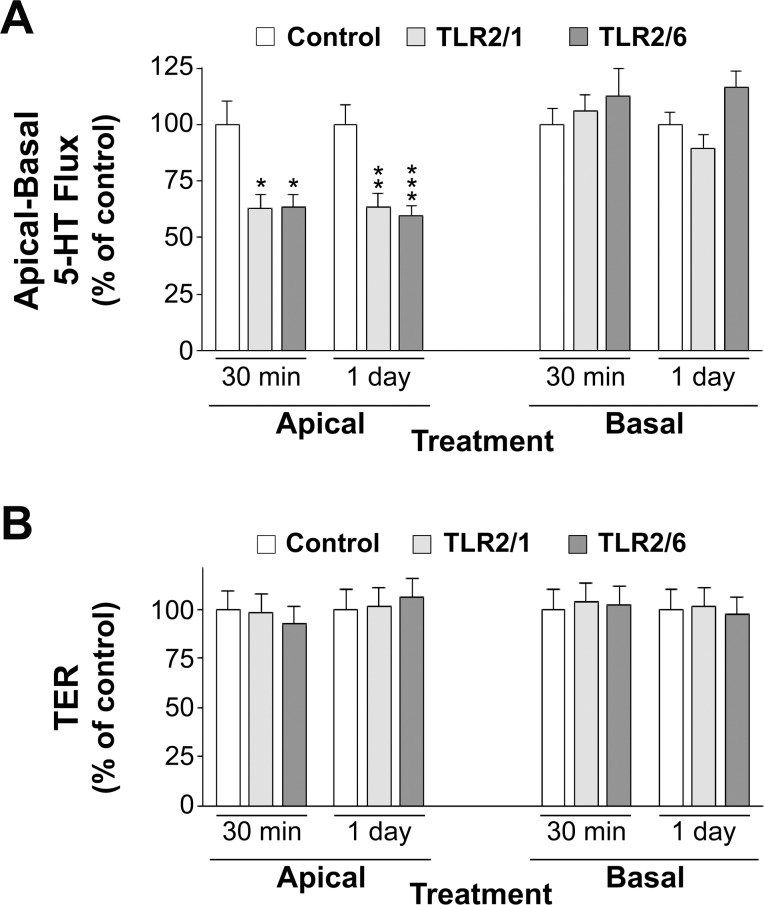
Effect of Pam3CSK4 (TLR2/1 ligand) and Pam2CSK4 (TLR2/6 ligand) on 5-HT transepithelial flux and resistance. (A) 5 μg/ml Pam3CSK4 or 50 ng/ml Pam2CSK4 were added to the apical or basal side for 30 min or 1 day and 5-HT apical-to-basal (A–B) flux was measured. The control condition corresponds to untreated cells. 5-HT concentration was 0.1 μM, and samples were taken every 10 min. The results are expressed as the percentage of the control value (100%) and are the mean ± SEM of 3 independent experiments. Absolute control values in pmol 5 HT/10 min were 0.53±0.06 and 0.63±0.06 (apical, 30 min, and 1-day treatment, respectively); 0.42±0.04 and 0.48±0.03 (basal, 30 min, and 1-day treatment, respectively). ***P<0.001, **P<0.01, and *P<0.05 compared with the control value. (B) Transepithelial resistance (TER) values under the same experimental conditions as in flux measurement are represented. Results are expressed as the percentage of control value (100%) and are the mean ± SEM of 3 independent experiments. Absolute control values in Ω/cm^2^ were 323.22±25.30 and 275.33±18.50 (apical, 30 min, and 1-day treatment, respectively), 301.56±31.07 and 280.50±30.10 (basal, 30 min, and 1-day treatment, respectively). No differences were observed.

The results reported above have shown that apical activation of TLR2 inhibits SERT activity in the apical membrane; however, whether basal TLR2 may act on apical SERT activity remained unknown. To assess this possibility, cells cultured in transwell plates were treated from either the apical or the basal compartment with 5 μg/ml Pam3CSK4 (TLR2/1 ligand) or 50 ng/ml Pam2CSK4 (TLR2/6 ligand) for 30 min and 1 day, and 5-HT apical to basal (A–B) flux was measured. As the results show, apical activation of TLR2/1 and TLR2/6 diminished significantly 5-HT A–B flux; however, basal activation of either TLR2/1 or TLR2/6 did not affect A–B flux (Fig 2A). The effect of TLR2 activation on transepithelial resistance was also analyzed in the cell monolayer under the same conditions as in the experiments of 5-HT fluxes measurement. The results have shown that TLR2/1 and TLR2/6 activation did not alter transepithelial resistance (Fig 2B).

### TLR2/1 and TLR2/6 activation decrease SERT mRNA and protein levels in Caco-2/TC7 cells

From the previous results, it may be inferred that TLR2 activation inhibits SERT activity. In order to gain an in-depth knowledge of this TLR2 effect, SERT expression was analyzed by measuring SERT mRNA and protein levels in Caco-2/TC7 cells treated for 30 min and 1 day with 5 μg/ml Pam3CSK4 or 50 ng/ml Pam2CSK4.

**Fig 3:**
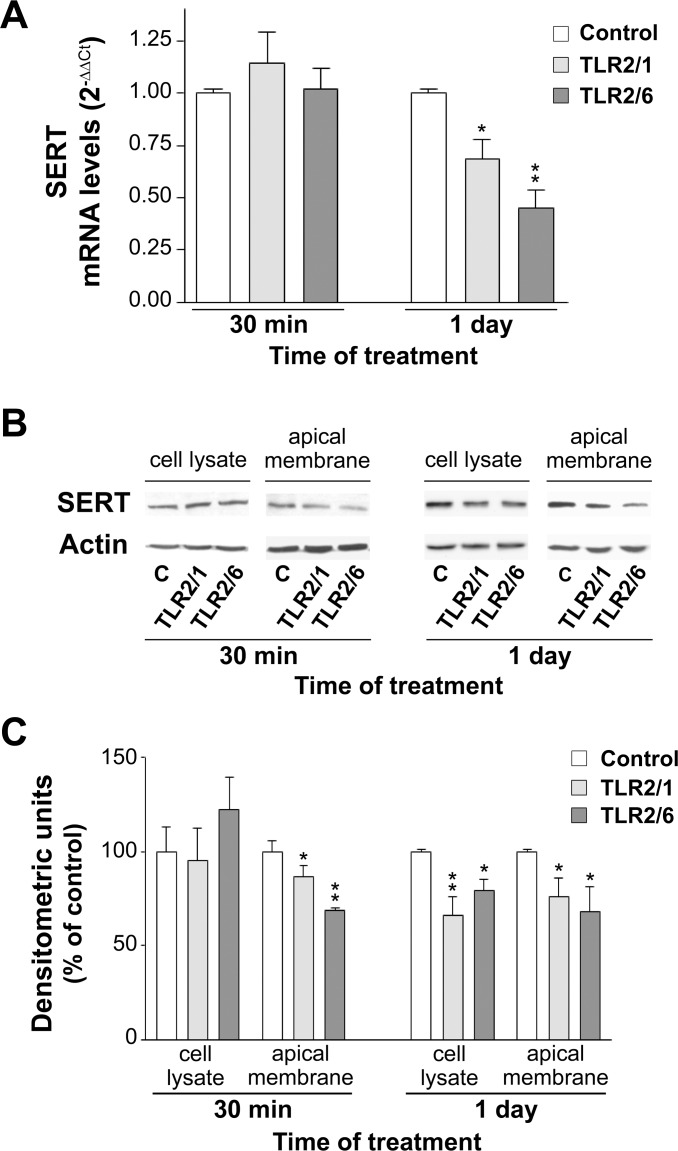
Effect of Pam3CSK4 (TLR2/1 ligand) and Pam2CSK4 (TLR2/6 ligand) on SERT mRNA and protein expression level. (A) Real-time PCR analysis of SERT mRNA expression level in cells treated for 30 min or 1 day with 5 μg/ml Pam2CSK4 or 50 ng/ml Pam3CSK4. Relative quantification was performed using comparative Ct method (2^–ΔΔCt^). Results are expressed as arbitrary units (control = 1) and are the mean ± SEM of 5 independent experiments. **P<0.01 and *P<0.05 compared with the control value. (B) Immunodetection of SERT by western blot in cell lysate and apical membrane from Caco-2/TC7 cells treated with 5 μg/ml Pam3CSK4 or 50 ng/ml Pam2CSK4 for 30 min or 1 day. (C) Quantitation of SERT protein in both cell lysate and apical membrane using β-actin as an internal control of the protein load (SERT/β-actin ratio). The results are expressed as a percentage of the control value and are the mean ± SEM of 5 independent experiments. **P<0.01 and *P<0.05 compared with the control value.

The results showed that long-term (1-day) activation of TLR2/1 or TLR2/6 decreased the SERT mRNA level; however, short-term (30 min) treatment did not seem to affect it (Fig 3A). The analysis of SERT protein level revealed that it was significantly diminished in the apical membrane of cells treated with TLR2/1 or TLR2/6 ligands either at short- or long-term periods. In contrast, SERT protein level in cell homogenate (total SERT) was only diminished after long-term treatment with either TLR2/1 or TLR2/6 ligands (Fig 3B and 3C).

### Analysis of SERT expression in *Tlr2*^*-/-*^ mice

**Fig 4:**
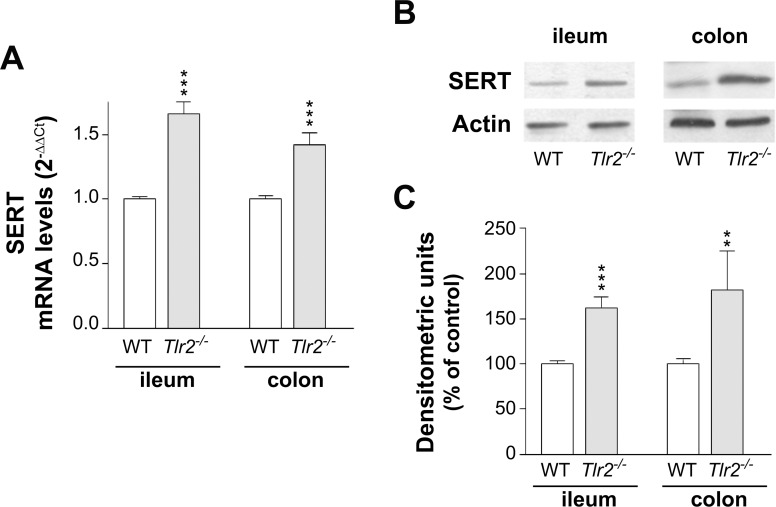
SERT mRNA and protein levels in the intestine from *Tlr2*^*-/-*^ mice. (A) Real-time PCR analysis of SERT mRNA expression level in ileum and colon. Relative quantification was performed using comparative Ct method (2^–ΔΔCt^). Results are expressed as arbitrary units (WT = 1) and are the mean ± SEM of 10 animals. ***P<0.001 compared with the control value. (B) Immunodetection of SERT by western blot in lysate from ileum and colon. (C) Quantitation of SERT protein in lysate from ileum and colon using β-actin as an internal control of the protein load (SERT/β-actin ratio). The results are expressed as a percentage of the control value (100%) and are the mean ± SEM of 10 animals. ***P<0.001 and **P<0.01 compared with WT.

To confirm the effect of TLR2 on SERT expression, SERT mRNA and protein levels in both ileum and colon of *Tlr2*^*-/-*^ mice were measured. As the results show, the levels of SERT mRNA (Fig 4A) and protein (Fig 4B and 4C) were significantly higher in the intestinal tract of *Tlr2*^*-/-*^ than in WT mice, suggesting that TLR2 may act as a repressor of SERT expression in the intestinal tract.

### Intracellular signaling pathways involved in the TLR2 effect on SERT activity

**Fig 5:**
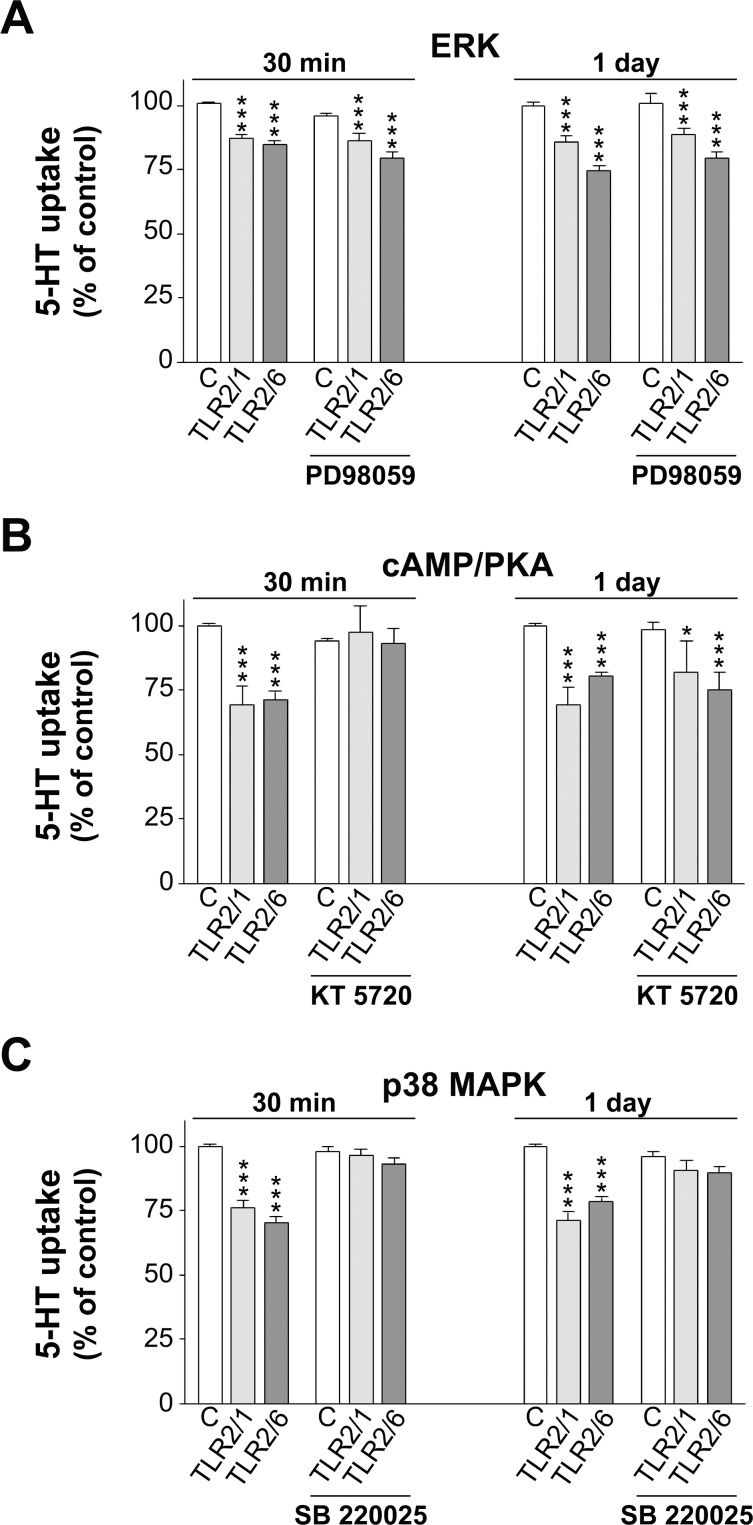
Intracellular mechanisms involved in Pam3CSK4 (TLR2/1 ligand) and Pam2CSK4 (TLR2/6 ligand) effect on SERT activity. Cells were treated for 30 min or 1 day with 5 μg/ml Pam3CSK4 or 50 ng/ml Pam2CSK4, and/or the different modifiers. Uptake of 5-HT was measured after 6 min of incubation, and 5-HT concentration was 0.2 μM. The results were compared with untreated cells (control). (A) ERK pathway. Cells were treated with Pam3CSK4, Pam2CSK4, and/or 40 μM PD98059. Absolute control values were 10.73±0.33 and 10.54±0.11 pmol 5-HT/mg protein for 30 min and 1 day, respectively. (B) cAMP/PKA pathway. Cells were treated with Pam3CSK4, Pam2CSK4, and/or 1 μM KT 5720. Control absolute values were 9.00±0.16 and 8.61±0.29 pmol 5-HT/mg protein for 30 min and 1 day, respectively. (C) p38 MAPK pathway. Cells were treated with Pam3CSK4, Pam2CSK4, and/or 1 μM SB 220025. Absolute control values were 7.11±0.26 and 7.93±0.12 pmol 5-HT/mg protein for 30 min and 1 day, respectively. The results, in all cases, are expressed as the percentage of the uptake control and are the mean ± SEM of four independent experiments. ***P<0.001, **P<0.01, and *P<0.05 compared with the corresponding control value.

In order to analyze TLR2 effects on SERT activity more in depth, the intracellular signaling pathways involved were assessed. To do so, 30 min before treatment of Caco-2/TC7 cells with either 5 μg/ml Pam3CSK4 or 50 ng/ml Pam2CSK4 (TLR2/1 and TLR2/6 ligands, respectively) during 30 min or 1 day, the cells were treated with or without different inhibitors of the pathways studied. Then, SERT activity was analyzed by 5-HT uptake measurement. Firstly, the ERK signaling pathway, which has been described as mediating TLR2 activity in epithelial cells [30], was studied. Caco-2/TC7 cells were treated with TLR2/1 or TLR2/6 ligands, with or without 40 μM PD98059, a selective inhibitor of the ERK pathway. The results showed that TLR2/1 and TLR2/6 effects on SERT were not reverted by PD98059 treatment, either at 30 min or 1 day treatment (Fig 5A). Therefore, ERK signaling pathway did not seem to be involved. Next, cAMP/PKA pathway was assessed by treating Caco-2/TC7 cells with TLR2/1 or TLR2/6 ligands, with or without 1 μM KT 5720, a selective PKA inhibitor, for 30 min or 1 day. The results obtained have shown that only short-term (30 min) TLR2/1 and TLR2/6 effects on SERT were reverted by KT 5720 (Fig 5B). Finally, p38 MAPK pathway was assessed; thus, Caco-2/TC7 cells were treated for 30 min or 1 day with TLR2/1 or TLR2/6 ligands, with or without 1 μM SB 220025, a selective inhibitor of p38 MAPK. The results show that SB 220025 reversed the effect of TLR2/1 and TLR2/6 on SERT at both short and long-term treatment (Fig 5C).

**Fig 6:**
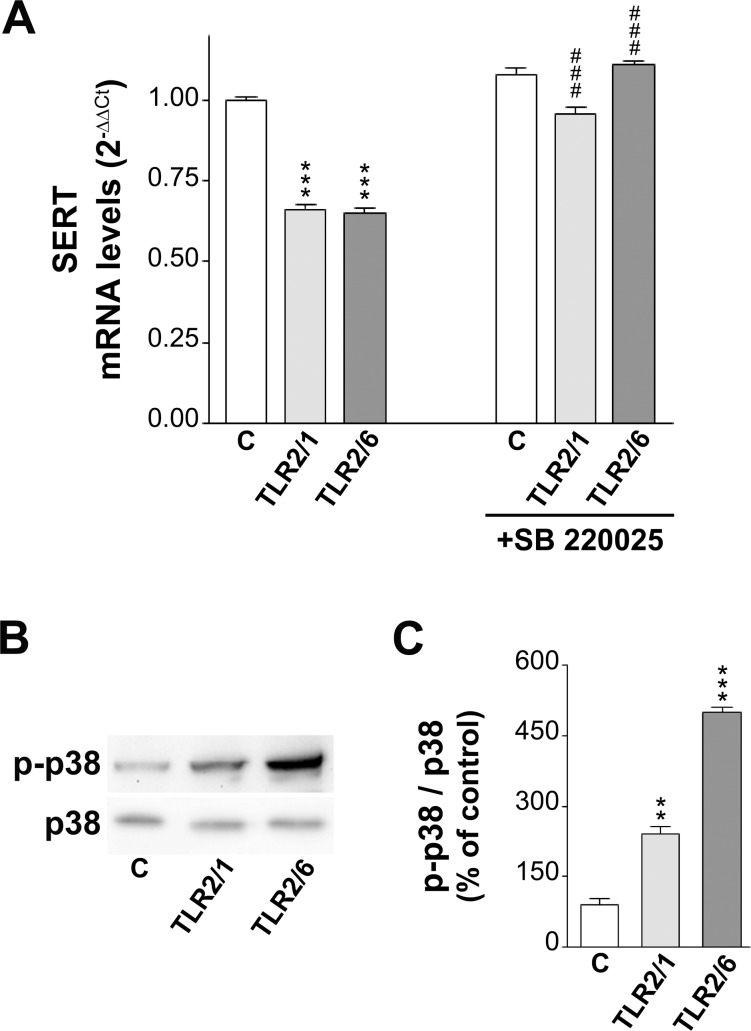
Involvement of p38 MAPK on TLR2 effect on SERT mRNA expression. (A) Real-time PCR analysis of SERT mRNA expression in cells treated with 5 μg/ml Pam3CSK4, 50 ng/ml Pam2CSK4, and/or 1 μM SB 220025. Relative quantification was performed using comparative Ct method (2^–ΔΔCt^). Results are expressed as arbitrary units (control = 1) and are the mean ± SEM of 5 independent experiments. ***P<0.001 compared with the control value; ^###^P<0.001 compared with corresponding values without SB 220025. (B) Immunodetection of p38 MAPK and p-p38 MAPK protein levels by western blot in cell homogenate of Caco-2/TC7 cells treated with 5 μg/ml Pam3CSK4 or 50 ng/ml Pam2CSK4. (C) Quantification of p-p38 MAPK and p38 MAPK relative expression in Caco-2/TC7 cells treated with Pam3CSK4 or Pam2CSK4. Results are expressed as p-p38 MAPK/p38 MAPK ratio and are the mean ± SEM of 4 independent experiments. ***P<0.001 and **P<0.01 compared with the control value.

The results obtained have suggested that long-term treatment (1 day) of Caco-2/TC7 cells with TLR2/1 and TLR2/6 ligands induces a reduction of SERT expression (mRNA and protein levels). In order to confirm the role of p38 MAPK pathway in long-term TLR2 effects, firstly, SERT mRNA level was measured by real-time PCR in cells treated for 1 day with TLR2/1 or TLR2/6 agonists with or without p38 MAPK inhibitor SB 220025. The results showed that the reduction of SERT mRNA induced by TLR2/1 and TLR2/6 ligands treatment disappeared when the cells were simultaneously treated with SB 220025. Moreover, SB 220025 alone did not appear to affect the level of SERT mRNA (Fig 6A). Secondly, as the phosphorylated form (p-p38 MAPK) mediates p38 MAPK activity, the levels of both p-p38 MAPK and p38 MAPK, and the ratio p-p38 MAPK/p38 MAPK were determined by western blot using specific antibodies. The results showed that long-term activation of both TLR2/1 and TLR2/6 induced an increase of the ratio p-p38 MAPK/p38 MAPK, suggesting the involvement of p38 MAPK pathway in long-term TLR2/1 and TLR2/6 effects (Fig 6B and 6C).

### 5-HT feedback regulation of TLR2 expression

**Fig 7:**
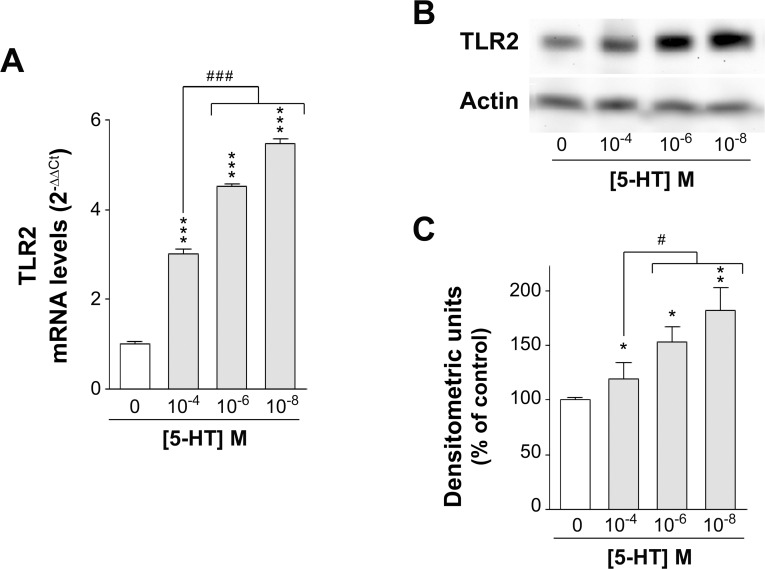
TLR2 mRNA and protein expression in 5-HT treated cells. (A) Real-time PCR analysis of TLR2 mRNA expression level. Relative quantification was performed using comparative Ct method (2^–ΔΔCt^). Results are expressed as arbitrary units (control = 1) and are the mean ± SEM of 4 independent experiments. ***P<0.001 compared with the control value; ^###^P<0.001 compared with 5-HT 10^−4^ M condition. (B) Immunodetection of TLR2 by western blot in cell lysate of Caco-2/TC7 cells treated with 10^−8^, 10^−6^, and 10^−4^ M 5-HT for 1 day. C: Quantification of TLR2 protein expression in cell lysate using β-actin as an internal control of the protein load (TLR2/β-actin ratio). The results are expressed as a percentage of the control value (100%) and are the mean ± SEM of 8 independent experiments. **P<0.01 and *P<0.05 compared with the control value. ^#^P<0.05 compared with 10^−4^ M 5-HT condition.

The results described above demonstrate that long-term TLR2/1 and TLR2/6 activation inhibit SERT activity and expression. This effect may trigger an increase of the 5-HT extracellular availability, which in turn might yield a feedback effect on TLR2 expression. In order to confirm this hypothesis, TLR2 mRNA and protein levels were measured in Caco-2/TC7 cells treated during 1 day with different 5-HT concentrations, simulating intestinal physiological (10^−8^ M) or inflammatory (10^−4^ M) conditions [31, 32]. A significant increase in the TLR2 mRNA (Fig 7A) and protein (Fig 7B and 7C) levels compared with the control (untreated cells) was found. This effect was higher at the lower 5-HT concentration (10^−8^ M), and was progressively decreasing as 5-HT concentration was increasing (Fig 7).

## Discussion

The intestinal activity of TLR2 is crucial for intestinal homeostasis [9, 10]. 5-HT, a key intestinal neuromodulator, has been found altered in inflammatory conditions [18, 20, 31]. As SERT is the major target to manage 5-HT levels, we studied the effects of TLR2 on intestinal SERT.

The present work has analyzed the effect of TLR2 activation, under the two-heterodimeric forms (TLR2/1 and TLR2/6) on intestinal SERT activity and expression. Reciprocally, the modulation of TLR2 molecular expression by 5-HT has also been assessed. The human enterocyte-like Caco-2/TC7 cells, which were used to carry out this study, have been previously shown to express SERT [27] as well as TLR2, TLR1, and TLR6 [1, 33]

We demonstrated that the activation of either TLR2/1 or TLR2/6 by specific ligands (Pam3CSK4 and Pam2CSK4, respectively) inhibits SERT activity. This effect of TLR2 was obtained at both short (30 min) and long-term (1-day) treatment of the cells by acting from the apical compartment, confirming the role of TLR2 as a transducer of microbiota apical information. The transepithelial resistance of the cellular monolayer did not seem to be affected by TLR2 activation. Therefore, monolayer paracellular permeability did not seem to interfere with the effect of TLR2 on 5-HT transport. This result is in agreement with a previous study suggesting that TLR2 stimulation maintains tight junction-associated barrier assembly of the intestinal epithelium [9].

TLR2 inhibition of SERT activity seems to be due to the diminution of the capacity of the transporter (*V*_max_), which may be the consequence of a reduction of SERT availability in the cell membrane. This result was confirmed by a decrease of SERT protein level in the apical membrane yielded by both TLR2/1 and TLR2/6 at short- and long-term treatment. Interestingly, TLR2/1 and TLR2/6 long-term treatment reduced SERT mRNA and cell lysate SERT protein levels, whereas short-term treatment did not appear to alter them. These results suggest that the activation of TLR2 at short-term treatment might reduce SERT expression by post-translational mechanisms; however, at long-term treatment, TLR2 might diminish SERT expression by acting on transcriptional or post-transcriptional processes.

It is clear that microbiota could play a key role in intestinal homeostasis, not only through activation of TLRs but also as a producer of metabolites as butyrate or serotonin. Thus bacteria could interfere directly on intestine homeostasis [34]. Previous results from our group demonstrated that serotonin has an inhibitory action on SERT [29], therefore, either through activation of TLRs or by their own serotonin, intestinal microbiota could inhibit SERT function, in turn increasing 5-HT levels.

SERT is considered a critical pharmacological target to control 5-HT levels, in fact, selective serotonin reuptake inhibitors (SSRIs) are being widely used in treatments for depression or mental disorders. SSRIs, in addition to their antidepressant effects, have been reported to have anti-inflammatory effects, being able to reduce pro-inflammatory cytokine levels in several inflammatory diseases as arthritis [35] or chronic colitis [36]. However SSRIs efficacy in IBD patients is still unclear [37, 38]. Fluoxetine and citalopram, two important SSRIs, have shown to inhibit the signalling of TLRs 3, 7, 8, and 9, providing a potential mechanism for their anti-inflammatory action [35]. Fluoxetine was also demonstrated to reduce LPS-induced pro-inflammatory IL-6 and TNF-α in human peripheral blood mononuclear cells [39]. However, the effect SSRIs on TLR2 remain unexplored, which would be an additional way of regulation of 5-HT levels.

The effect of Pam3CSK4 and Pam2CSK4 is considered to be mainly due to the activation of TLR2; however, a TLR2-independent effect cannot be discarded. To confirm the role of TLR2 on SERT expression, both ileum and colon from *Tlr2*^*-/-*^ mice were used to measure the level of SERT mRNA and protein. The results showed that SERT expression in the intestinal tract was significantly higher in *Tlr2*^*-/-*^ than in WT mice, corroborating our results obtained *in vitro*. From these results, it might be inferred that TLR2 may act as a repressor of intestinal SERT expression. The results obtained in the intestinal tract from *Tlr2*^*-/-*^ mice are assumed to be due to TLR2 deficiency; however, alteration of expression of another TLRs cannot be dismissed as it has been recently reported in macrophages [40].

Further, we intended to investigate the underlying intracellular pathways. Firstly, the involvement of ERK was analyzed since recent studies carried out in neuronal cells [30] and macrophages [41] have demonstrated that this pathway mediated TLR2 effects. However, the results obtained have shown that ERK pathway did not seem to mediate TLR2 effects in our cell model. Following this analysis, the role of the cAMP/PKA pathway was analyzed since it has been stated that it is involved in the immune response of TLR2 [42] and in SERT regulation [43]. Our results suggest that cAMP/PKA may mediate TLR2/1 and TLR2/6 effect, but only at short-term treatment. A previous study has demonstrated that cAMP/PKA system inhibits SERT activity in intestinal epithelial cells by post-translational regulation [27], which may confirm the involvement of cAMP/PKA in TLR2 short-term effect. Finally, p38 MAPK pathway, which has been demonstrated to mediate TLR2 activity in different cell types [44, 45], was analyzed. The results have shown that p38 MAPK seemed to mediate TLR2/1 and TLR2/6 effect on SERT at both short and long-term treatment. The involvement of p38 MAPK in the regulation of SERT has been shown to be controversial; thus, in rat brain, p38 MAPK did not appear to affect SERT activity [46], and, in contrast, results in neural cells have suggested that p38 MAPK enhances SERT activity [47]. Our results agree with a previous study in intestinal epithelial cells, in which p38 MAPK activation seemed to mediate SERT activity inhibition [24].

TLR2 has been shown to be able to discriminate triacylated from diacylated lipopeptides by the heterodimerization with TLR1 or TLR6, respectively. Our results show that both TLR2/1 and TLR2/6 yielded an inhibitory effect on SERT by triggering the same mechanism. These results may suggest a redundant effect of TLR2 in intestinal epithelial cells; this feature of TLR2 has also been found in other cell types [48], although recent results have concluded that TLR2/1 and TLR2/6 could exhibit specific signaling transduction [49]. In relation to the ability shown by TLR2 to inhibit SERT activity and expression by different pathways depending on the period of treatment, it might be considered an advantageous way of specialization to modulate 5-HT in different situations. Since TLR2 has been described to activate additional cellular signaling pathways that have not been analyzed in the present work [9], their mediation in the effects on SERT cannot be fully discarded.

In agreement with our results, previous studies carried out in intestinal epithelial cells have shown that several TLRs are able to regulate intestinal SERT activity by different mechanisms. Thus, TLR4 activation by LPS inhibited SERT activity by posttranscriptional mechanisms involving PKC [23], and TLR3, which is an intracellular TLR activated mainly by viral components, was also shown to modulate SERT activity by activating the p38-MAPK pathway [24]; however, in this case, TLR3 did not affect SERT molecular expression. Moreover, a recent study has demonstrated that TLR2 may regulate the expression of 5-HT receptors in intestinal tract [50]. These results confirm the essential role of intestinal microbiota in the regulation of intestinal serotoninergic system by acting through different microorganism-associated molecular patterns.

Our results show that TLR2 activation inhibits SERT and, consequently, it may induce an increase of extracellular 5-HT availability in the intestine, which in turn might regulate TLR2 expression in the intestinal epithelium by a negative feedback mechanism. The results have shown that 5-HT, at any concentration tested, increased TLR2 expression, with maximum effect at physiological concentration and gradually reduced as 5-HT concentration was increased. This result demonstrates a feedback regulation between TLR2 and 5-HT and suggests that 5-HT under physiological conditions may maintain an elevated level of TLR2 expression to guarantee TLR2 activity as a microbiota transducer. However, under high 5-HT concentration, which is a condition that occurs in inflammatory bowel diseases [18], the level of TLR2 expression decreases, thus reducing TLR2 responses. Previous studies have inferred that a prolonged and excessive activation of TLRs can lead to uncontrolled inflammation in the host [51]. Our results demonstrate that 5-HT, an intestinal non-immunological modulator, down-regulates TLR2 expression, which added to different pro-inflammatory effects concurring in inflammatory processes may contribute to modulation of the intestinal response to inflammation.

Overall, the results of the present work demonstrate for the first time that TLR2, under both heterodimeric forms TLR2/1 and TLR2/6, inhibits intestinal SERT activity and expression. This effect seemed to be mediated by cAMP/PKA and p38 MAPK pathways that might trigger post-translational and/or post-transcriptional regulation of SERT expression. In addition, 5-HT feedback regulation of TLR2 has also been demonstrated. Previous studies support the TLR2 contribution to the intestinal homeostasis [9]. Moreover, recent results reveal that TLR2 seems to be essential for the development of inflammation [10] and oncogenesis [52]. From our study the involvement of TLR2 in both, intestinal homeostasis and intestinal pathology, may be inferred, not only by its inherent innate immune role, but also by regulating the intestinal serotoninergic system. The present work may also clarify intestinal serotoninergic responses induced by the microbiota. Understanding host-microbiota interaction in the intestinal tract may provide new insights into intestinal homeostasis and inflammation.

## Supporting Information

S1 Fig Kinetic study of SERT activity after TLR2 activation. The cells were treated during 30 min or 1 day with 5 μg/ml Pam3CSK4 or 50 ng/ml Pam2CSK4. The 5-HT range concentration was 0.05–5 μM. The uptake conditions are described in Material and Methods. The results are the mean of 4 experiments.
(TIF)
