# Supplementary figures and images for: Intestinal Serotonin Transporter Inhibition by Toll-Like Receptor 2 Activation. A Feedback Modulation
Source: PLoS One. 2016 Dec 29;11(12):e0169303. doi: 10.1371/journal.pone.0169303 (PMC5199115; doi:10.1371/journal.pone.0169303)

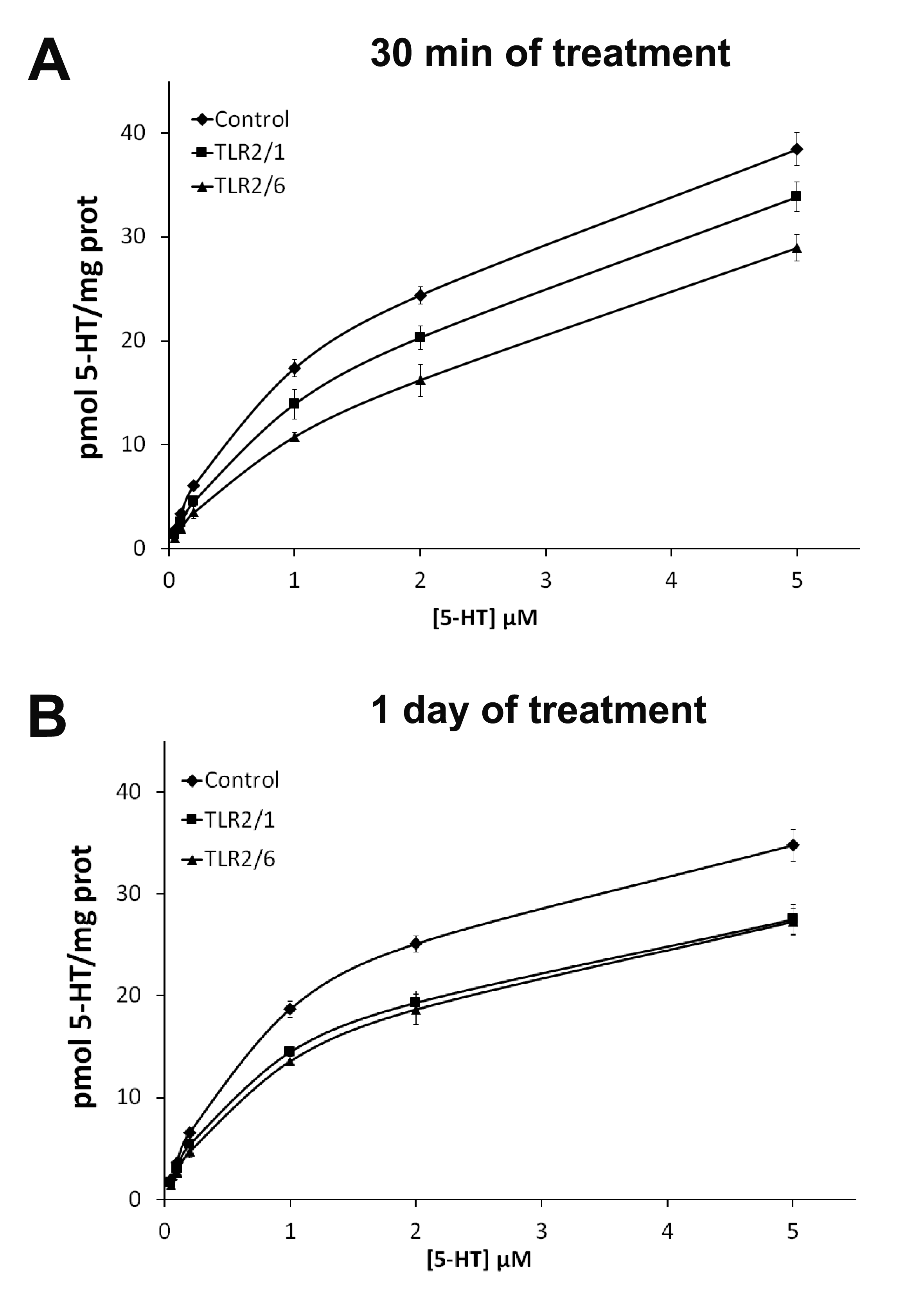

Supplement: S1 Fig — The cells were treated during 30 min or 1 day with 5 μg/ml Pam3CSK4 or 50 ng/ml Pam2CSK4. The 5-HT range concentration was 0.05–5 μM. The uptake conditions are described in Material and Methods. The results are the mean of 4 experiments. (TIF) [file pone.0169303.s001.tif]
